# Straight to the Point—The Novel Strategies to Cure Pediatric AML

**DOI:** 10.3390/ijms23041968

**Published:** 2022-02-10

**Authors:** Monika Lejman, Izabela Dziatkiewicz, Mateusz Jurek

**Affiliations:** 1 Laboratory of Genetic Diagnostics, II Faculty of Pediatrics, Medical University of Lublin, A. Gębali 6, 20-093 Lublin, Poland; 2 Student Scientific Society, Laboratory of Genetic Diagnostics, II Faculty of Pediatrics, Medical University of Lublin, A. Gębali 6, 20-093 Lublin, Poland; izabeladziatkiewicz@gmail.com (I.D.); mateusz.jurek777@gmail.com (M.J.)

**Keywords:** AML, pediatric, targeted therapy, CAR-T

## Abstract

Although the outcome has improved over the past decades, due to improved supportive care, a better understanding of risk factors, and intensified chemotherapy, pediatric acute myeloid leukemia remains a life-threatening disease, and overall survival (OS) remains near 70%. According to French-American-British (FAB) classification, AML is divided into eight subtypes (M0–M7), and each is characterized by a different pathogenesis and response to treatment. However, the curability of AML is due to the intensification of standard chemotherapy, more precise risk classification, improvements in supportive care, and the use of minimal residual disease to monitor response to therapy. The treatment of childhood AML continues to be based primarily on intensive, conventional chemotherapy. Therefore, it is essential to identify new, more precise molecules that are targeted to the specific abnormalities of each leukemia subtype. Here, we review abnormalities that are potential therapeutic targets for the treatment of AML in the pediatric population.

## 1. Introduction

Acute myeloid leukemia (AML) is a relatively rare heterogeneous group of hematologic malignancies in children, but it causes disproportionate mortality [1]. AML is associated with various molecular alterations in myeloid stem cells leading to rapid and uncontrolled growth and differentiation arrest of leukemic cells in bone marrow (BM) [2]. Despite the fact that outcome has improved over the past decades due to improved supportive care, a better understanding of risk factors, and intensified chemotherapy, pediatric AML remains a life-threatening disease, and overall survival (OS) remains near 70%. The outcome depends mainly on molecular and cytogenetic aberrations, and thus on the initial response to treatment. The majority of AML cases represent de novo entities, but there is also evidence of AML as a secondary malignancy [3]. The incidence rate of AML in infants, individuals aged 1–4, and individuals aged 5–9 is respectively 1.5, 0.9, and 0.4 per 100,000 individuals per year, while in adulthood it gradually increases up to an incidence of 6.12 per 100,000 elderly individuals (over 80 years old) [4]. Epidemiological studies indicate that AML is one in five leukemias occurring in children and one in three leukemias occurring in adolescents and young adults. Therefore, age is considered as a very strong prognostic factor, independent of other risk factors [5,6]. Pediatric AML diagnosis schema includes methods such as cytochemistry, immunophenotyping, morphology, and molecular genetics [4]. The curability of AML is due to the intensification of standard chemotherapy, more precise risk classification, improvements in supportive care, and the use of minimal residual disease to monitor response to therapy. Chemotherapy offers a good chance of recovery in acute promyelocytic leukemia and in a form with specific favorable molecular features. In other forms of acute myeloid leukemia (AML), the cure rate with chemotherapy alone is very low (10–15%). The use of very intensive chemotherapy with auto-HCT increases the chance of a cure to about >40%, and allo-HCT to about >60% of patients. Nevertheless, due to the high proportion of patients with unfavorable prognostic factors, the treatment results in the entire group of unfavorable AML risk are still poor. Therefore, it is essential to identify new, more precise molecules that are targeted to the specific abnormalities of each leukemia subtype [7]. Recent research shows potential for on-target/off-tumor immunotherapeutic toxicity due to target antigen expression on non-malignant cells [8].

## 2. Genetic Subtypes and Characterization

The development of genetics, whole genome, exome, and RNA sequencing in recent years has significantly contributed to a better understanding of the molecular landscape of AML. Although many of these studies were conducted using adult AML cases, the range of mutations observed in childhood AML is similar, but differences are observed in the frequency of these changes in childhood cases [9,10]. According to the type of cell from which the leukemia has developed and its degree of maturity, AML is divided into eight subtypes (M0–M7), which are the French-American-British (FAB) classification. FAB classification takes into account the appearance of the malignant cells using light microscopy and/or cytogenetics to characterize underlying chromosomal abnormalities and thus immunophenotype [5]. As previously mentioned, age is an important prognostic factor, because the frequency of cytogenetic subgroups of AML is associated with age, and different subtypes vary in responses to therapy. There is evidence of an association of age with the cytogenetic profile of AML; increasing age is associated with a decrease in favorable and an increase in unfavorable cytogenetic changes (Table 1) [11,12]. Differences in adult and pediatric AML genetics are shown in Table 2.

## 3. Genetics Influence the Success of Treatment

### 3.1. Low-Risk Genetics

Alterations associated with a favorable prognosis occur about once in ten cases of childhood AML, such as translocation (8;21), chromosome 16 inversion, *NPM1* and *CEBPA* gene mutations [24].

#### 3.1.1. *RUNX1-RUNX1T1*

Non-random chromosomal aberration t(8;21)(q22;q22) is one of the best-known mutations; it usually correlates with the AML M2 subtype and results in *RUNX1::RUNX1T1* fusion gene formation, which was one of the first fusion genes to be used for minimal residual disease (MRD) monitoring [65]. t(8;21) occurs in approximately 10–12% of childhood AML cases [13]. Morphologically, this aberration is commonly associated with a relatively low WBC count (10,000/μL) and the presence of large blasts with abundant basophilic cytoplasm containing plenty of azurophilic granules and Auer rods. There have also been cases of blasts with very large granules, which could indicate a fusion of these cells. In contrast, smaller blasts containing t(8;21) aberration could be found in the peripheral blood and promyelocytes, myelocytes, and mature granulocytes with abnormal nuclear segmentation and/or distinctive homogenous pink colored cytoplasm could be found in the bone marrow. In addition to this, in the cohort with t(8;21)(q22;q22) and inv(16)(p13.1;q22), infants are extremely rare and 5-year OS is roughly at the 80–90% level [13,14]. Treatment of AML patients with mutation t(8;21) is based mainly on the use of anthracyclines and cytarabine, followed by 2–4 cycles of cytarabine. Therapy can be assisted by the addition of gemtuzumab-ozogamicin (GO), which is an anti-CD33 antibody [66]. Recently, the Japanese Paediatric Leukaemia/Lymphoma Study Group (JPLSG) conducted a trial (protocol number AML-05) on a group of 100 *RUNX1::RUNX1T1*–positive pediatric acute myeloid leukemia (AML) patients to determine risk factors for relapse. The results of the study indicate that CD19 negativity might be a distinct characteristic of the poor prognostic subgroup of *RUNX1::RUNX1T1*–positive AML patients. CD19-negative patients showed inferior RFS. Moreover, the results of a recent preclinical study suggest that the proteasome inhibitor bortezomib and the BCL-2 inhibitor ABT-737 can induce apoptosis and inhibit cell proliferation of *RUNX1::RUNX1T1* cells in vitro and in vivo [67].

#### 3.1.2. *CBFβ::MYH11*

Chromosome inv(16)(p13;q22)or t(16;16)(p13.1;q22), which in the majority of cases is associated with M4 subtype of pediatric AML with a specific abnormal eosinophil component (M4Eo), is a chromosome aberration that leads to a fusion gene *CBFB-MYH11* formation. *MYH11* and *CBFB* encode the smooth muscle myosin heavy chain (SMMHC) and β-subunit of core binding factor (CBF), respectively, and the fusion protein resulting from inv(16)(p13;q22) contains 1–5 CBFβ exons and C-terminal region of SMMHC. It is known that oncogenic fusion protein CBFβ::SMMHC blocks the differentiation of myeloid cells and, therefore, is a potent inhibitor of leukemogenesis [14,68]. Patients with inv(16)(p13;q22) show a high WBC level (46,000/μL), excess of monocytes and characteristic abnormal eosinophil component, and are thus commonly called the M4Eo AML subgroup. Bone marrow contains eosinophils at all stages of maturation without significant maturation arrest; most striking is the fact that at the promyelocyte and myelocyte stages, these cells contain immature eosinophilic granules. The differences between immature and mature granules are that immature ones are often larger, purple-violet in color, and in some of cells so dense that they obscure the cell morphology [69]. AML with t(8;21) or inv(16) are usually reported together as core binding factor AML (CBF-AML) [49]. CBF-AML accounts for approximately 25% of pediatric and 15% of adult de novo AML patients, and it is considered as the most common cytogenetic subtype of AML [70]. OS for pediatric patients with CBF-AML compared to AML with normal cytogenetics is slightly better, but a subset of these patients has a poor prognosis, suggesting heterogeneity in this patient population and indicating that additional mutational changes may influence disease pathogenesis [71,72]. Ro5-3335 is a benzodiazepine that blocked the interaction between CBFβ and RHD in preclinical studies in mice. Ro5-3335 binds both RUNX1 and CBFβ and inhibits the activity of RUNX1. It is possible that Ro5-3335 causes a conformational change in either RUNX1 or CBFβ that does not block heterodimerization but alters the complex’s DNA binding specificity or its ability to activate and/or repress transcription. In leukemic mice, Ro5-3335 reduces disease burden and increases survival. Extended treatment of wildtype mice with Ro5-3335 causes minor changes in platelet and red blood cells [68].

#### 3.1.3. Mutated *NPM1* without *FLT3/ITD*

Cytogenetically normal AML (CN-AML) aberrations can also be classified as alterations associated with a favorable prognosis, and these include *NPM1* and *CEBPA* gene mutations [73]. The *NPM1* gene, which is localized at chromosome 5q35, encodes nucleophosmin, which is predominantly localized in the nucleoli but shuttles rapidly between the nucleus and cytoplasm, and its function differs depending on cellular processes. Nucleophosmin is involved in centrosome duplication through cyclin E/cyclin-dependent kinase 2 (CDK2) phosphorylation, ribosome biogenesis, maintenance of genomic integrity, preventing aggregation of proteins in the nucleolus, and also in the ARF-p53 tumor-suppressor pathway [16,74,75]. Mutations in exon 12 of *NPM1* occur much more frequently in adult compared to pediatric AML, or 50–60% to about 10% of cases, respectively [24,58,76,77]. There are several *NPM1* mutation variants, but all of them consist of an insertion in the C-terminal region, causing aberrant movement of the protein to the cytoplasm (NPMc+), which is present in approximately one-third of adult AML cases [78]. AML with NPMc+ is highly associated with *FLT3*/ITD (internal tandem duplication) mutations, high WBC, a higher percentage of blasts, and most importantly, with improved response to chemotherapy induction, but better long-term outcomes and OS were observed in *NPM1* mutations without *FLT3*/ITD mutation, also in the pediatric population (5-year OS of 85%) [16,17,79,80]. The standard of care for adult patients with *NPM1* mutations is chemotherapy. A specific feature of AML cells with *NPM1* mutation is high expression of CD33; therefore, such cases can be treated with gemtuzumab ozogamicin. Adding this agent to chemotherapy improved EFS and OS. In clinical practice, allo-HSCT transplant is recommended in patients with *NPM1* mutation with *FLT-3* and not recommended for patients with *NPM1* mutation without *FLT-3*. Inducing nucleolar stress could be therapeutically effective in *NPM1*-mutated AML. Actino-mycin D, which triggers nucleolar stress by inhibiting RNA polymerase I, induces CR in *NPM1*-mutated AML. Another therapeutic approach that may be effective in treating AML is blocking *NPM1* oligomerization, because pentameric/decameric *NPM1*_wt_ is required for proper nucleolus formation. According to other studies, targeting *HOX* expression may turn out to be effective in this AML [81,82,83].

#### 3.1.4. *CEBPA*

Mutations in the *CEBPA* gene, which encodes CCAAT/enhancer binding protein alpha (C/EBPα), occurs in about 2.4% and 5.6% of childhood AML patients (for single and double mutants, respectively) and shows favorable prognosis with 80% 5-year OS for double mutants vs. 25% for single mutants [18,24]. C/EBPα is one of the essential transcription factors responsible for myeloid cell development; therefore, *CEBPA* gene mutations cause a selective early block of granulocyte differentiation [84]. Moreover, *CEBPA* promoter’s hypermethylation, resulting in *CEBPA* silencing, is one of the mechanisms that give an effect similar to that of mutation [85]. It has also been shown that C/EBPα function can be disrupted by post-transcriptional or post-translational inhibition by several oncogenes, for example, *FLT3*/ITD, *AML1-ETO* and *CBF-MYH11* [86,87]. According to scientific research, the AML subtypes with the *CEBPA* mutation show high sensitivity to the effects of treatment targeting *MLL1* histone-methyltransferase complex. *CEBPA*-mutated hematopoietic progenitor cells are hypersensitive to pharmacological targeting of the *MLL1* complex. Furthermore, the use of CRISPR/Cas9 to induce mutagenesis results in proliferation arrest and myeloid differentiation. The identification of *CEBPA_dm_* status in AML has major clinical importance, allowing relapse risk to be stratified properly for post-remission treatment. [88,89].

#### 3.1.5. *PML::RARA*

Translocation t(15;17)(q24.1;q21.2) resulting in *PML::RARA* fusion gene formation is the most common mutation driving the development of acute promyelocytic leukemia (APL), which is classified as FAB-M3 AML [15,90]. APL accounts for only 5–10% of pediatric AML cases and is found in about 2% of infants with AML [11]. RARA and PML encode retinoic acid (RA) receptor alpha and promyelocytic leukemia protein, respectively [91]. Nowadays, due to enormous improvement of therapy, APL outcomes are significantly better than a few decades ago, with an average OS near 95% and EFS of 90% [92,93]. The current standard of care for children with APL remains ATRA (all-trans retinoic acid) plus ATO (arsenic trioxide) in combination with chemotherapy. ATRA was introduced into treatment about 30 years ago. ATRA’s mechanism relies on binding to PML::RARα and induces a conformational change leading to the degradation of the fusion protein. Combination of ATRA with chemotherapy and ATO induces apoptosis in APL cells, yielding good results [90]. Analysis of treatment results of 20 pediatric patients treated in Australia showed 100% molecular remission (MR). Analysis of outcome (median follow up = 2.5 years) showed an OS of 63% and 73%, with and without abandonment, respectively. By risk group, the high-risk (HR) group (38% and 50% with and without abandonment, respectively) and standard-risk (SR) group (82% and 88% with and without abandonment, respectively) showed better outcomes [94]. However, clinical trials currently under way aim to reduce the use of chemotherapy. Treatments for relapsed patients mainly include GO, an anti-CD33 antibody. Recently, much attention has been directed to tamibarotene. Tamibarotene is a synthetic retinoid that inhibits proliferation and induces differentiation of malignant cells by binding to the retinoic acid receptor α/β, which shows a higher binding affinity for PML::RARα. In 2018, the final results of a prospective randomized JALSG-APL204 study were published in Nature. In this study, the authors compared tamibarotene with all-trans retinoic acid (ATRA) in the maintenance therapy for newly diagnosed acute promyelocytic leukemia (APL). Additionally, they reported the results of this study with a median follow-up of 7.3 years. A total of 269 patients in molecular remission who had received ATRA and chemotherapy were randomized into two groups: 135 to ATRA (45mg/m^2^ daily), and 134 to tamibarotene (6mg/m^2^ daily) for 14 days every 3 months for 2 years. The 7-year RFS was 84% in the ATRA arm and 93% in the tamibarotene arm (*p* = 0.027, HR = 0.44, 95% CI, 0.21 to 0.93). Tamibarotene has been shown to be more effective than ATRA in reducing relapses in high-risk patients [95].

### 3.2. High Risk Genetic

In addition to the genetic changes that are associated with a good prognosis and, therefore, a good response to treatment, there are also chromosome aberrations and gene mutations that lead to a poor outcome. Some of these are FLT3/ITD mutation, 11q23 rearrangements, t(5;11) (leads to *NUP98::NSD1*) or inv(16)(p13.3q24.3) (leads to *CBFA2T3::GLIS2*) [24]. In the case of 11q23 rearrangements, outcome depends mainly on partner genes that form fusion genes together with *KMT2A*. *MLLT3* and *MLLT11* together with *KMT2A* are associated with a good prognosis in pediatric AML, whereas the predicted outcome for *AFDN*, *MLLT10* and *ABI1* as partner genes is poorer [28].

#### 3.2.1. *FLT3*/ITD Mutation

*FLT3* proto-oncogene is an FMS-like tyrosine kinase 3 located in the chromosomal region 13q12.2. *FLT3* is involved in the proliferation, differentiation, and survival of cells during hematopoietic processes mainly in lymphohematopoietic organs—for example, BM, lymph nodes, liver, thymus and spleen, where it has the highest expression [96]. The major form of *FLT3* mutation is an ITD (internal tandem duplication) in exons 14 and 15, which leads to ligand-independent auto-phosphorylation and activation of the receptor [97]. *FLT3*/ITD mutation occurs relatively often in childhood AML; it occurs in about 10–20% of pediatric AML cases, but the frequency increases with age, from 1.5% in infants to 7% in children aged 1–5 years to nearly 17% in adolescents and young adults. In contrast to alterations with a favorable prognosis, the 5-year OS is 30–40% for patients with high allelic ratios, which means a high mutant to normal allelic ratio [24,25]. Additionally, *FLT3*/ITD is an important therapeutic target, and despite that mutation of this gene leads to a poor prognosis. Rapid *FLT3*/ITD diagnosis allows early intervention with targeted therapies [82]. Sorafenib is a multikinase inhibitor that targets *FLT3*/ITD mutations and has a key role in tumor cell signaling, proliferation, and angiogenesis [98]. Early-phase clinical trials in children with relapsed AML demonstrated that sorafenib was tolerable and effective when given with chemotherapy [99]. Another report from the Children’s Oncology Group (Protocol AAML1031) showed that Sorafenib improved rates of induction II CR, as well as 3-year EFS and reduced RR from CR, compared to historical controls [100]. The effectiveness of sorafenib also has been confirmed in clinical trials involving adult AML patients. The efficacy of *FLT3*/ITD inhibitor has been studied at 15 centers in Germany and Austria. Authors reported data from a randomized, placebo-controlled, double-blind phase II trial (SORMAIN). Adult patients with *FLT3*/ITD–positive AML (*n* = 83) in complete hematologic remission after HCT were randomly assigned to receive for 24 months either the multitargeted and FLT3-kinase inhibitor sorafenib (*n* = 43) or placebo (*n* = 40). The results showed that sorafenib maintenance therapy reduces the risk of relapse and death after HCT for patients with confirmed *FLT3*/ITD mutation [101].

#### 3.2.2. 11q23/*KMT2A* Rearrangements

Some of the most frequent chromosome aberrations in AML are *KMT2A* gene (also known as the *MLL* gene) rearrangements, which are located in the chromosomal region 11q23 [102]. The *KMT2A* gene, which encodes a histone 3 lysine 4 methyltransferase, involved in the regulation of transcription and epigenetic modulations, has at least 77 fusion partners, and most of the rearrangements lead to the formation of fusion proteins, and because of that, the prognostic impact depends on the specific recombination [29]. 11q23/*KMT2A* rearrangements occur in 20% of childhood AML cases and most frequently in infants [24]. The most frequent 11q23/*KMT2A* abnormality, occurring in 6–9% of pediatric patients, is t(9;11)(p22;q23), resulting in fusion of *KMT2A* with the *MLLT3* gene, which presents a better outcome compared with any other 11q23/*KMT2A* rearrangement both in adult and pediatric AML. *MLLT3* is the most common partner, representing approximately 50% of all pediatric AML cases with *KMT2A* rearrangements, and this subtype is associated with an intermediate prognosis [27,28,102]. There are also other subtypes representing intermediate prognosis, such as t(11;19)(q23;p13) either with the *ELL* gene (19p13.1) or *MLLT1 (ENL)* gene (19p13.3), which account for 1–2% of all AML pediatric patients [29,30]. Translocation t(1:11)(q21;q23), resulting in *KMT2A-MLLT11* fusion, also leads to favorable clinical outcomes, but there are some 11q23/*KMT2A* rearrangements that, independently of other prognostic factors, result in a poor prognosis, such as t(10;11)(p12;q23) and t(6;11)(q27;q23), which involve *MLLT10* and *MLLT4* as fusion partners for *KMT2A*, respectively. *MLLT10* is more common among infants (2–3% of all pediatric AML cases), whereas *MLLT4* is prevalent in older children (1–2% of all pediatric AML cases) [30]. There is also t(10;11)(p12;q14), which leads to *PICALM::MLLT10* fusion, a rare (<1% of all pediatric AML cases) abnormality assigned to an intermediate risk group and that can closely resemble t(10;11)(p12;q23) in the FISH analysis [21]. Another fusion partner for *KMT2A* is *AFF1* (AF4/FMR2 family member 1), which encodes a member of the AF4/lymphoid nuclear proteins related to the Fragile X E syndrome (FRAXE) family of proteins. *KMT2A::AFF1* fusion is rarely found in AML, but it is also known to be a molecular marker of infant acute lymphoblastic leukemia (ALL) [103]. In addition, KMT2A fusion proteins also recruit the DOT1L histone 3 lysine 79 (H3K79me) methyltransferase that positively regulates the expression of critical target genes. The histone methyltransferase DOT1L is involved in supporting the proliferation of MLL-r cells, for which a target inhibitor, Pinometostat, has been evaluated in a clinical trial recruiting pediatric MLL-r leukemic patients. Targeting DOT1L with Pinometostat sensitizes pediatric AML cells to further treatment with the multi-kinase inhibitor Sorafenib. It causes an increase in apoptosis and growth suppression of both AML cell lines and primary pediatric AML cells with diverse genotypes [104].

#### 3.2.3. 11p15/*NUP98::NSD1* Rearrangements

Another chromosomal aberration associated with unfavorable outcomes in childhood AML is t(5;11)(q35;p15), occurring in approximately 3–4% of cases [24,31]. This translocation results in *NUP98::NSD1* fusion gene creation, where *NUP98* is nucleoporin 98-kDa and *NSD1* is nuclear receptor-binding SET-domain protein 1 [105]. Translocation t(5;11) is characterized by 4-year event free survival (EFS) below 10% [32]. It is well-known that this aberration is frequently found along with deletion of the long arm of chromosome 5; moreover, *NUP98::NSD1* also has a strong association with previously mentioned *FLT3*/ITD mutation [31,33]. *NUP98::NSD1*-positive AML is often associated with other mutations similar to *FLT3*/ITD, *NRASG12D*, or *MYC*. Translocation t(11;12)(p15;p13) is another cytogenic abnormality involving the *NUP98* gene, which fuses with the *KDM5A* gene. This rearrangement, initially described in M7 pediatric AML, occurs in 2% of all childhood cases, and it is associated with a poor prognosis with 5-year OS rate of around 33% [34,35,36]. Sagarajit Mohanty et al. [106] analyzed the synergistic effect of *NUP98::NSD1* and *NRASG12D* using an in vivo mouse model. To demonstrate the leukemic potential of *NRASG12D*, *NUP98::NSD1*, and *NUP98::NSD1 + NRASG12D* in vivo, they transplanted transduced mouse bone marrow cells into mice. The authors reported that mice transplanted with *NRASG12D* transduced cells did not show any engraftment over 11 months. *NUP98::NSD1* mice showed very low engraftment, while *NUP98::NSD1* + *NRASG12D* mice showed rapidly increasing engraftment over 12 weeks. Moreover, mice that had been transplanted with *NUP98::NSD1* cells developed leukemia with long latency, and mice transplanted with *NUP98::NSD1+NRASG12D* cells developed aggressive leukemia with short latency. The median survival was 251 vs. 54 days after transplantation, *p* = 0.001. Reported data might suggest that targeted inhibition of the fusion might be a potential treatment in *NUP98::NSD1*-positive AML patients. This appears to be a promising area for further clinical research [106]. In other preclinical studies, Johannes Schmoeller et al. [107] investigated the effectiveness of CDK4/CDK6 inhibition in in vivo and in vitro models of *NUP98*-fusion AML. The authors revealed CDK6 as a highly expressed, directly regulated target of *NUP98*-fusion proteins. Palbociclib, an inhibitor of CDK4/CDK6 that is approved for breast cancer therapy, was tested on both *NUP98*-rearranged cell lines and mice transplanted with *NUP98::NSD1* leukemia cells. Monotherapy using a patient-derived xenograft model of *NUP98::NSD1*-rearranged AML caused a highly significant prolongation of survival compared to the vehicle-treated control cohort (median survival 152 vs. 103 days). Collected data show that NUP98-rearranged AML is sensitive towards CDK4/CDK6 inhibition in vivo and in vitro [107].

#### 3.2.4. *MNX::ETV6*

One of the most common chromosome aberrations in pediatric AML is t(7;12)(q36;p13); it is present in about 30% of infant AML cases and is associated with a poor outcome [108]. This translocation involves heterogenous breakpoints at 7q36 of the *MNX1* gene (previously named *HLXB9*) and 12p13 of the *ETV6* gene, which is a motor neuron and pancreas homeobox 1, and ETS variant transcription factor 6, respectively [108,109,110]. The majority of t(7;12) cases have been reported in high association together with coexisting aberrations, e.g., additional copies of chromosomes 8, 19 and/or 22 [111]. Due to specific translocation breakpoints at the chromosomal level (breakpoints affecting the terminal regions of both chromosomes 7 and 12), t(7;12)(q36;p13) is considered as a cryptic rearrangement, and the specific transcript is present in approximately 50–60% of patients [109]. It is reported that 3-year EFS for pediatric patients with t(7;12) is below 24% [24,37].

#### 3.2.5. Aberrations Involving *GATA2* and *MECOM* (*EVI1*)

Inversion inv(3)(q21q26.2) or translocation t(3;3)(q21;q26.2) are abnormalities that share the same chromosomal breakpoints; both involve *GATA2* and *MECOM* genes, and both result in relocation of *GATA2*’s enhancer to the vicinity of *MECOM*. As a result of these abnormalities, *GATA2* is silenced, and *MECOM* is overexpressed [112]. In adult AML these aberrations are well-known, but in childhood AML they occur in 1–2% of cases as a poor prognosis lesion (long-term OS < 10%), with a median age of 3 years. There are several coexisting secondary abnormalities associated, such as monosomy 7, dysmorphic platelets and megakaryocytes, and high platelet count [11,40,41].

### 3.3. Intermediate Risk

Among many chromosomal and genetic abnormalities in pediatric AML, there is still a subgroup of alterations whose presence, on the one hand, for example, may cause significant regression of the disease, but, on the other hand, may negatively impact response to therapy.

#### 3.3.1. *MYST3::CREBBP*

One of the examples of alterations of uncertain significance is t(8;16)(p11;p13), whereby *MYST3* gene encoding a histone acetyltransferase is fused with *CREB-binding protein (CREBBP)* gene encoding nuclear receptor coactivator. Both proteins are involved in transcriptional regulation and cell cycle control [46,113]. The mentioned translocation is observed in 10% of childhood AML cases [24]. This change is interesting because several cases of spontaneous remission in AML patients with t(8;16) have been observed and described in the literature. Characteristic features of patients with this aberration were bluish papular rash at the time of birth and an increased leukemic blast count in BM [45,46]. t(8;16) patients’ morphology meets the criteria for myelomonocytic (M4) or monocytic (M5) FAB type AML [114]. One case showed spontaneous remission 4 months after initial diagnosis, and complete remission at least 11 months long [45].

#### 3.3.2. Trisomy 21

Children with Down syndrome (DS) have a substantially increased risk of multiple health conditions. They have a particularly elevated risk (estimated 150-fold) of developing AML before age 5. AML-affected children develop a unique type of malignancy, referred to as myeloid leukemia of Down syndrome (ML-DS), which is recognized as a separate entity in the actual World Health Organization (WHO) classification of leukemia. Approximately 15% of pediatric AML cases occur in DS children. ML-DS demonstrates unique characteristics such as the predominance of FAB M7, an age predilection during the first 4 years of life, and higher sensitivity to chemotherapeutic agents, which translate into a good treatment response as well as increased treatment-related toxicities. [22]. AML in DS children is associated with several unique features. There is a high prevalence of the acute megakaryocytic leukemia (AMKL) phenotype 2. Moreover, a mutation in the gene for the X-linked transcription factor GATA1 occur in almost all DS patients. The most frequent imbalances in ML-DS are duplications in 1q (16%), or deletions in 7p (10%) and/or 16 (7.4%) [23]. The cytogenetic profiles of ML-DS cases differ significantly from non-DS patients with AML [16,18,19]. ML-DS children show more frequently acquired trisomies of chromosomes 8, 11, and 19, dup(1p), del(6q), del(7p), dup(7q), and del(16q). Typically, the favorable translocations associated with non-DS AML (e.g., t(8;21); t(15;17); inv(16), 11q23 rearrangements) are rarely seen in ML-DS patients. For ML-DS children older than 4 years, cytogenetic features, molecular biology findings and response to therapy significantly diverge from younger patients [115]. Among the secondary molecular abnormalities, we can distinguish mutations in cohesion complex genes: *STAG2, RAD21, MPL, RAS, JAK2, JAK3* [116].

#### 3.3.3. *KIT* Mutations

*KIT* is a protooncogene that encodes transmembrane glycoprotein, which is one of the type III receptor tyrosine kinase family members. KIT, upon binding with a stem cell factor, activates signaling pathways affecting proliferation, differentiation, and survival of hematopoietic stem cells. Mutations in exons 8, 10, 11 and 17, which encode extracellular, transmembrane, juxtamembrane domains and activation loop of the tyrosine kinase domain, respectively, lead to ligand-independent activation of KIT [111]. These mutations are considered as a prognostic factor in the adult CBF-AML population and may be associated with worse clinical outcomes [47,117]. The clinical significance in the pediatric CBF-AML population is less clear; however, it is estimated that incidence is at the level of 5%, and 25% of patients have a favorable prognosis, but on the other hand, *KIT* mutations may negatively impact response to therapy; therefore, *KIT* remains a factor of uncertain significance [47,48].

#### 3.3.4. *FLT3/TKD*

Mutations among tyrosine kinase domain of *FLT3* gene (*FLT3*/TKD) have an incidence of 7% in childhood inv(16) AML, which is significantly less than in adult patients with the aforementioned inversion, where it accounts for 28% of cases [24,49]. In the study conducted by N. Duployez et al., the *FLT3*/TKD mutations in CBF-AML patients with a mutant allelic ratio of 10% or greater were associated with a higher cumulative incidence of relapse (CIR) compared with a lower ratio and non-mutated patients. The 5-year CIR was 58.8%, 20.0% and 31.5%, respectively [49]. Findings of *KIT* and *FLT3*/TKD mutations highlight the multiclonality of CBF-AML and encourage investigators to delve deeper into the topic and advance the science in this area so that better identification of risk in AML patients will be possible.

#### 3.3.5. *BCR::ABL1*

*BCR::ABL1* fusion gene, resulting from Philadelphia chromosome formation, is one of the most characteristic features of chronic myeloid leukemia (CML), and it has also been found in AML [113]. In 2016, WHO published the WHO classification of myeloid neoplasms and acute leukemia, and *BCR::ABL* + AML was listed there as a provisional entity. For AML to be classified as *BCR::ABL* + AML, patients with de novo AML must not show any evidence of underlying CML and aberrations such as mutated *NPM1* or *CEBPA*, t(9;11)(p21.3;q23.3), t(8;21)(q22;q22.1), or inv(16), and inv(3) must not be present, as in this case leukemia would be classified as “AML with recurrent genetic abnormalities” [43,114]. According to European Leukemia Net (ELN) [40] and current National Comprehensive Cancer Network guidelines [115] *BCR::ABL* + AML is classified as a disease with a poor outcome [40,115]. Therefore, we believe that *BCR::ABL* + AML is classified as a high-risk disease because if the previously mentioned aberrations coexisted, the disease could not be so classified. It seems that the prediction of *BCR::ABL* + AML prognosis is much more complicated and depends mainly on specific genetic background, such as coexisting aberrations, rather than *BCR::ABL* itself.

#### 3.3.6. *NPM1::MLF1*

Another aberration with uncertain significance for prognosis is t(3;5)(q25;q35), which results in the formation of chimeric gene *NPM1::MLF1*, where *NPM1* and *MLF1* encode nucleophosmin and myelodysplasia/myeloid leukemia factor 1, respectively [42,116]. This aberration is mainly described in young adults, and in the case of pediatric patients (mainly M2, M4 and M6), its frequency is below 0.5%; thus, prognosis prediction is complicated and risk at diagnosis remains intermediate [42,53].

#### 3.3.7. t(16;21)

There are two distinct abnormalities important for pediatric AML that are related to translocation between 16 and 21 chromosomes: t(16;21)(p11;q22) and t(16;21)(q24;q22), which produce fusion proteins *FUS::ERG* and *RUNX1::CBFA2T3*, respectively. t(16;21)(p11;q22) occurs in about 0.4%, and the second one in 0.2% of pediatric AML cases; therefore, prognosis prediction is complicated, but the I-BFM Study Group indicated that the prognosis for *FUS::ERG* is poor with 4-year EFS of 7%, and outcome for *RUNX1::CBFA2T3* is significantly better with 4-year EFS of 77% [20,21].

#### 3.3.8. *RBM15::MKL1*

Another chromosome aberration that shows an intermediate outcome is translocation t(1;22)(p13;q13). This translocation occurs only in infants and young children, with a median age of 0.7 years, and in general in 0.3% of all pediatric AML cases, mainly in the AMKL cohort [35,50,51]. This abnormality leads to fusion of the *RBM15* and *MKL1* genes, and clinically it manifests as abnormal megakaryopoiesis, as this is characteristic for the FAB M7 subtype of AML [35]. Patients carrying this entity have intermediate outcome with a 5-year EFS of 54.5% and 5-year OS of 58.2% [50].

#### 3.3.9. Trisomy 8

Trisomy 8 in pediatric AML may occur either as a sole cytogenetic change or it can be associated with another chromosomal aberration, and thus frequency significantly differs—trisomy 8 is generally present in about 10–14% of pediatric AML patients, but only in 3% of cases as a sole abnormality and mainly over the age of 10 [13,54]. The most frequent co-existing abnormalities are *FLT3*/ITD*, KMT2A* rearrangements and trisomy 19, 6 and 21. Prognostically, trisomy of chromosome 8 seems to be associated with intermediate or poor prognosis, but no molecular data were provided, and a poor prognostic impact seems to be mainly dependent on the coexisting aberrations; thus, we categorize it as an intermediate/discussed aberration (5-year EFS ~25%) [54,55].

#### 3.3.10. Monosomy 7/5 or Del(5q)

Abnormalities of chromosome 5q, primarily deletions, both in adult and pediatric AML cohorts, lead to a higher WBC and blast count at diagnosis [38,118]. The prevalence of monosomy 5 or del(5q) accounts for 1.2% of pediatric AML cases, of which 61.5% are male patients and the median age is 12.5 years. The 5-year OS and 5-year EFS are 27% and 23%, respectively [38]. Monosomy 7 occurs at a rate of 4% of AML cases in children with a median age of 5.5 years. Patients with monosomy 7 show an inferior outcome with 10-year OS of 32% and 10-year EFS of 29% [13].

#### 3.3.11. Hyperdiploid and Complex Karyotypes

A hyperdiploid karyotype can be defined as three or more numerical gains of chromosomes. According to the NOPHO-AML trial, which included 596 pediatric patients with AML, 11% were hyperdiploid cases with 48–65 chromosomes. The most frequent solely numerical gains were trisomies of 6, 8, 21 and 19. Clinically, these cases were not shown to have a poor prognosis, but on the other hand, were strongly associated with AMKL and lower WBC count [56,57]. Complex karyotype, as described in BFM98 trial analysis, can be defined as three or more chromosomal aberrations, including at least one structural chromosomal aberration and excluding favorable cytogenetics and *KMT2Ar*, and was found in 8% of pediatric AML cases as a poor risk factor [14]. In another study, namely the MRC trial, the definition of complex karyotype did not exclude *KMT2Ar*, but it was detected in 15% of pediatric AML cases and showed an intermediate prognosis [13].

### 3.4. Mutations That May Significantly Affect Prognosis

Among the variety of genetic changes outlined above, there are some somatic mutations with well-established prognostic relevance, such as *NPM1, FLT3/*ITD*, CEBPA_dm_* and *WT1_mt_*, that can significantly affect the prognosis of pediatric AML patients when they coexist with other mutations and aberrations. These anomalies were presented in Table 3. The presence of these mutations can improve a patient’s prognosis or, independently of other genetic factors, significantly worsen it.

*NPM1* encodes nucleophosmin and is translocated or mutated in several hematologic malignancies, forming a variety of fusion proteins, such as NPM::ALK, NPM::RARa, NPM::MLF1, or NPM mutant products [118]. Nucleophosmin plays several key roles in the cell life cycle; it is involved in ribosome biogenesis, apoptotic response to stress and oncogenic stimuli. It maintains genomic stability by controlling DNA repair mechanisms and stabilizes the oncosuppressor ARF and determines its subcellular localization, which leads to growth pathway suppression [52,119,120,121,122]. *NPM1* along with *CEBPA_dm_* are mainly found in normal karyotype cases, assigning them to a low-risk category. It is also worth noting that the coexistence of *FLT3*/ITD with *NPM1* mutations counteracts its negative influence on prognosis [12,16,123]. *CEBPA_dm_* is significantly associated with *GATA2* mutations, *FLT3*/ITD, and *CBFB::MYH11*, and shows a positive impact on OS [110,123].

*FLT3*/ITD, as mentioned above, is the major form of *FLT3* gene mutation [90]. Because of its frequent occurrence with childhood AML, *FLT3*/ITD co-occurs with a normal karyotype and a variety of genetic aberrations, both those associated with good and poor prognosis, but also those for which the prognosis is intermediate. Examples of secondary cytogenetic abnormalities for *FLT3*/ITD are as follows: t(15;17)(q24.1;q21.2), t(8;21)(q22;q22), inv(16)(p13.1q22) or t(16;16)(p13.1;q22), t(5;11)(q35;p15), t(6;9)(p22;q34), which lead to formation of *PML::RARA, RUNX1::RUNX1T1, CBFB::MYH11, NUP98-NSD1*, and *DEK-NUP214*, respectively, and furthermore trisomy 8, *CEBPA_dm_* and mutated *WT1* [13,14,54]. In most cases, *FLT3*/ITD is a bad prognostic factor, and it worsens the outcome, which is equal to poorer OS and/or EFS [123].

The *WT1* gene is known to be overexpressed among leukemias including childhood AML. Mutated *WT1* is often found to be a secondary aberration along with *CEBPA* and *NPM1* gene mutations, *N-RAS*, and *K-RAS*, and it has a strong association with *NUP98::NSD1* and *FLT3/*ITD [12,123]. *WT1_mt_*, similar to *FLT3*/ITD, worsens the outcome [110].

**Table 3:** Significant modifiers of prognosis in pediatric AML.

Molecular Alteration	Most Common Secondary Cytogenetic Factors	Influence on Prognosis	References
*NPM1* gene mutations	*FLT3/*ITD Normal karyotype	Improve the prognosis	[12,16,123]
*CEBPA* gene mutations	*FLT3/*ITD *GATA2* mutations *CBFB::MYH11* Normal karyotype	Improve the prognosis	[12,16,110,123]
*FLT3/*ITD	Normal karyotype *PML::RARA* *RUNX1::RUNX1T1* *CBFB::MYH11* *NUP98::NSD1* *DEK::NUP214* trisomy 8 *CEBPA_dm_* mutated *WT1*	Generally, worsens the prognosis with a few exceptions (e.g., *NPM1*)	[13,14,54,123]
Mutated *WT1*	*NUP98::NSD1* *FLT3*/ITD *CEBPA* *NPM1* *N-RAS* *K-RAS*	Worsens the prognosis	[12,110,123]

Abbreviations: AML: acute myeloid leukemia; ITD: internal tandem duplication.

## 4. New Therapeutic Achievements

### 4.1. Immunotherapy

The development of immunotherapy for the treatment of AML in the pediatric population faces many barriers. The main one is the lack of an antigen specific only to cancer cells. Furthermore, AML blasts create an immunosuppressive microenvironment. Due to the fact that the majority of surface proteins that define malignant myeloid blasts are also expressed on normal progenitors, potential therapeutic targets are mainly seen in dysregulated gene expression [8]. However, it should be noted that the impaired expression of immunotargets significantly differs between adults and children. For example, immunotargets of adult AML, such as IL3RA, were not overexpressed in pediatric AML. The best described antigen AML tumor associated antigen (TAA) is the sialic acid-binding immunoglobulin lectin (SIGLEC) CD33 [7,124]. It has been proven that this antigen is found on most AML and progenitor cells. GO is a humanized anti-CD33 antibody that shows activity in pediatric and adult patients with AML [125]. A recently conducted single-center, phase III, double-arm trial (AAML0531) enrolled 1022 children, adolescents, and young adults aged 0 to 29 years with newly diagnosed AML. Patients were randomly assigned to either standard five-course chemotherapy alone (Arm A) or to the same chemotherapy with two doses of GO (3 mg/m^2^/dose) administered once in induction course 1 and once in intensification course 2 (Arm B). Data obtained showed GO significantly improved EFS (3 years: 53.1% vs. 46.9%; hazard ratio [HzR], 0.83; 95% CI, 0.70 to 0.99; *p* = 0.04) but not OS (3 years: 69.4% vs. 65.4%; HzR, 0.91; 95% CI, 0.74 to 1.13; *p* = 0.39) [126]. Collected data showed that GO added to chemotherapy improved EFS through a reduction in RR for children and adolescents with AML. JL1 is a CD43 epitope and cell surface glycoprotein of the mucin family, which is expressed during lymphoid maturation but is not expressed on mature blood cells. Recent studies reported that JL1 antigen is expressed on leukemic T, B, and myeloid lineage cells in >80% of acute leukemia patients and thus could serve as a potential candidate for immunotherapy. In a recent clinical trial conducted in Korea, authors included 78 patients diagnosed as having de novo pediatric acute leukemia (52 ALL and 26 AML) with a median age of 96 months (range: 2–216 months) and a median follow-up period of 424 days (range: 79–753 days). JL1 expression assessment was performed by flow cytometry, and positive JL1 expression was defined as ≥ 20% expression among the gated leukemic blasts. The study demonstrated that de novo pediatric AML patients with positive JL1 expression have higher CD13 and lower CD65 and CD15 expressions than patient without JL1 expression. Moreover, it was noted that de novo pediatric AML patients with positive JL1 expression presented with *RUNX1::RUNX1T1*, *CBFB::MYH11*, and *PML::RARA* rearrangements, which lead to chromosomal aberrations. These results suggest that JL1 may be a potential therapeutic target in immunotherapy for pediatric AML patients [127]. In the context of AML immunotherapy, it is worth approximating the results of a clinical trial with the use of flotetuzumab. Flotetuzumab (MGD006) is an investigational bispecific antibody-based molecule to CD3e and CD123 engineered in a DART format. CD3-engaging molecules work by stimulating the effector cells of the immune system in order to inactivate cancer cells. Knowing that high CD123 expression is also associated with a poor prognosis, flotetuzumab targeting of CD123 represents an interesting treatment option [128].

**Figure 1:**
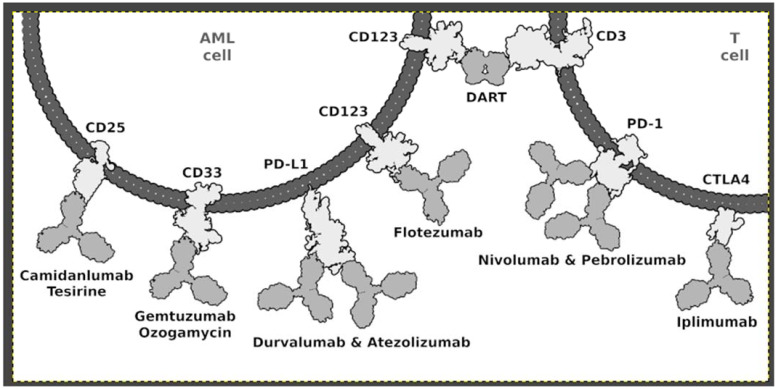
Monoclonal antibodies and their inhibitory targets on the surfaces of AML cells and T cells. Abbreviations: AML: acute myeloid leukemia; DART: dual-affinity re-targeting.

In recent years, research has identified the engagement of immune checkpoint receptors as a mechanism of tumor evasion. T-cell checkpoint receptors such as CTLA-4 and PD-1 relay inhibitory signals that modulate T-cell activation. In acute myeloid leukemia, PD1 expression is observed on T-cell subpopulations, including CD4+ effector T cells, CD4+ Treg, and CD8+ T cells, both in untreated patients and in relapses. Increased PD1 expression on CD8+ T lymphocytes may be one of the factors leading to the dysfunction of this group of immune cells and a reduction in the immune response to the progressive course of AML. Blocking signaling through checkpoint receptors results in increased T-cell activation, with effector T-cell proliferation and increased cytotoxicity toward cancer cells [129]. Inhibitors of PD-1 (nivolumab) and CTLA-4 (ipilimumab) have shown promise for the treatment of advanced melanoma and relapsed Hodgkin’s lymphoma with response rates ranging from 7 to 40%. In vitro studies have shown that AML may utilize the PD-1/PD-L1 axis to evade an anticancer immune response. For adult cancer, inhibitors of PD-1 (nivolumab) and CTLA-4 (ipilimumab) have shown promise with response rates ranging from 7 to 40%. PD-1 and/or PD-L1 are expressed in AML cells, and their blockade coupled with the depletion of regulatory T cells showed potent anti-leukemic activity in preclinical models. Several monoclonal antibodies (e.g., Nivolumab, Prembrolizumab, Durvalumab, and Ipilimumab) are currently studied for their anti-leukemic potential in refractory/relapsed AML patients [103]. Nivolumab is a human anti-PD1 IgG4 monoclonal antibody that blocks its interaction with PDL1 and PDL2 [8]. Pembrolizumab, also known as MK3475, is a humanized IgG4 monoclonal antibody that binds to PD1, blocking its interaction with PDL1 and PD L2 ligands (Figure 1) [7].

### 4.2. CART-T

Therapy with T cells expressing chimeric antigen receptors that are specific for tumor antigens turned out to be a success in the treatment of patients with B-cell ALL [130]. That is why CAR-T therapy remains a highly promising strategy also for AML patients. The key to the success of this therapy is the identification of specific antigens for the cancer cells. The ideal antigen target should play a key role in cell differentiation and survival. In practice, determining such a therapeutic target is extremely difficult. AML cells express a variety of stem cell and myeloid differentiation antigens on the cell membrane, such as CD33, CD34, CD123, CD135. However, the same antigens are expressed on healthy bone marrow cells, causing normal hematopoiesis to be affected during treatment [131]. CD33 is expressed on about 85–90% of AML blast cells, making it a promising therapeutic target. Data obtained from preclinical studies support the effectiveness of an anti-CD33 CAR-T therapy for AML in mice [132,133,134]. Kim et al. [135] demonstrated an approach to prevent damage to physiological hematopoiesis. They produced CD33 knockout human hematopoietic stem cells and progenitor cells (HSPCs) that have been successfully implanted in immunodeficient mice. Edited donor allogeneic hematopoietic stem cells are not eliminated by anti-CD33 CART, which would efficiently eliminate leukemia cells without marrow toxicity. CD123 is expressed at the levels both of leukemic stem cells (LSCs) and more differentiated leukemic blasts [135,136]. Numerous preclinical studies have confirmed the efficacy of anti-CD123 CART in vivo and in vitro [137,138,139]. A novel approach to the subject was demonstrated by Simon Loff et al. [140], who presented data from the preclinical and translational development of a UniCAR-based treatment of acute leukemia. They showed efficient tumor reactivity in vitro and in vivo using T cells that were engineered to express a UniCAR construct optimized for clinical applications and redirected against CD123^+^ leukemia cells. UniCar technology has been designed so that T cells do not express any characteristic antigen. Instead, they express the universal CAR (UniCAR-T) that recognizes a small linear peptide derived from the nuclear human La/SS-B protein (UniCAR epitope (UCE)). UniCART-T remains inactive until it connects with targeting modules (TMs) consisting of the UCE linked to an appropriate binding domain. The UniCAR-Ts, in combination with TM123 effectiveness and safety, will be assessed in a clinical trial [140]. The use of CD123-targeting T cells could be an encouraging strategy for the potential treatment of AML patients. Currently (referring to the clinicaltrials.gov database [141]), one anti-CD123 CAR-T trial is being conducted. This is a phase 1 study. Subjects will receive CART123 cells via a single IV infusion at a dose of 2 × 10^6^ CART123 cells/kg, following lymphodepleting chemotherapy. The total dose administered to each subject will be based on the subject’s body weight obtained at the time of apheresis. The minimum acceptable dose for infusion is 1 × 10^5^ CART123 cells/kg [141]. In conclusion, the main problem of potent, antigen-specific immunotherapy for AML is the absence of truly AML-specific surface antigens, which pose a high toxicity risk. MGD006 is a bispecific CD3 × CD123 dual-affinity re-targeting (DART) molecule that binds T lymphocytes and cells expressing CD123, an antigen up-regulated in several hematological malignancies including AML. MGD006 mediates blast killing in AML samples, together with concomitant activation and expansion of residual T cells. In a preclinical study, Gurunadh R. et al. [142] provided preclinical activity, safety, pharmacokinetic, and pharmacodynamic data supporting MGD006, a CD3 × CD123 bispecific DART capable of redirecting host T cells to kill CD123^+^ targets, as a potential therapeutic agent for the treatment of CD123^+^ hematological malignancies [142].

### 4.3. Other Therapeutical Achievements

#### 4.3.1. CPX-351 (Vyxeos^®^)

CPX-351 (Vyxeos^®^) is a dual-drug liposomal encapsulation of cytarabine and daunorubicin that was rationally designed to improve efficacy over the traditional 7 + 3 cytarabine/daunorubicin chemotherapy regimen for patients with acute myeloid leukemia (AML). The CPX-351 liposome protects cytarabine and daunorubicin from metabolism and elimination. Thanks to this solution, the difference in the pharmacokinetics of both compounds is cancelled, and they can act simultaneously. In clinical studies, these liposome properties markedly increased the elimination half-life of CPX-351 versus free cytarabine and daunorubicin and maintained a synergistic drug ratio for over 24 h after administration. The use of a liposome allows for less exposure to tissues that are off-target tissues. CPX-351 shows high efficiency in patients with newly diagnosed high-risk/secondary AML [143].

#### 4.3.2. HDAC Inhibitors

HDACs catalyze the removal of acetyl functional groups from the lysine residues of the histones. HDACs may also play a role in the regulation of the immune system by targeting the transcriptional regulator STAT3. HDACs are important proteins; they directly regulate gene expression and control cellular activity by reversing the state of histone acetylation. If the chromatin structure is altered through (de)acetylation of histones, this may result in decreased or increased gene transcription, altering gene expression levels. Numerous scientific studies indicate that HDAC deregulation may lead to the development of neoplasms, including hematological neoplasms [144]. HDAC inhibitors can be classified most commonly into five groups: hydroxamates, benzamides, cyclic tetrapeptides, aliphatic acids, and electrophilic ketones. These inhibitors have shown the ability to induce differentiation, cell cycle arrest, and apoptosis in AML. However, preliminary preclinical studies suggest that HDAC inhibitors could be effective in combination therapy and not as monotherapy. Preclinical studies conducted on leukemic cell lines have shown that JAK2/HDAC dual inhibitors have therapeutic potential in treating AML [145].

## 5. NGS—Predisposition in Pediatric AML

An important approach in medicine is not only the treatment of a disease entity, but also the assessment of the predisposition for the development of the disease, even before its occurrence. The World Health Organization’s latest leukemia classification scheme has included germline predisposition to myeloid malignancies as a provisional category. Predictive testing has become possible since the widespread use of next-generation sequencing (NGS). The development of NGS techniques, commercially available since 2006, allowed for cost- and time-effective sequencing [146]. The data presented by A. Andersson et al. demonstrate that mutations in isocitrate dehydrogenase 1 (*IDH1*) and 2 (*IDH2*) are exceedingly rare in pediatric ALL, but are more common in pediatric AMLs, occurring in 3.5% of cases overall, and in 9.8% of pediatric AMLs with a normal karyotype. In their pediatric cohort, they could not demonstrate any significant statistical association of *IDH1/IDH2* mutations with overall survival or event-free survival, although the power of this analysis is influenced by the low overall frequency of *IDH1*/*IDH2* mutations [147]. In myeloid cancers, *IDH1/2* mutation has been identified as an induction event. However, *IDH1* mutations are mainly involved in early occurrences of AML. Mondesir et al. reported that *IDH1* modifications are found in about 10% of AML patients and are associated with worse outcomes in patients undergoing thorough chemotherapy [148]. Drazer et al. used NGS-targeted panels including genes associated with hereditary hematopoietic malignancies (HHMs) to identify pathogenic germline variants in malignant cells, thereby identifying patients at risk for HHMs. In total, pathogenic or likely pathogenic variants in *ANKRD26*, *CEBPA*, *DDX41*, *ETV6*, *GATA2*, *RUNX1*, or *TP53* were identified in 74 (21%) of 360 patients. Three *DDX41* variants, 2 *GATA2* variants, and a *TP53* variant previously implicated in Li-Fraumeni syndrome were of germline origin [149]. According to the latest research, *IDH2* mutations may occur in the early stages of AML leukemia development in children; their presence makes cells more susceptible to oncogenic activities of *FLT3* activating mutations. It makes them a potential gene that can be studied by NGS. Germinal mutations are one of the factors conditioning the development of hematological neoplasms that gives 100% certainty of tumor development regardless of environmental conditions. An example of one such mutation is a germline 5′-end *CEBPA* mutation. In addition, variants identified in leukemia cells should be considered as likely germline genes that can be mutated in germline or somatic tissues, including *TP53*, *CEBPA*, *RUNX1* and *DDX41* [150].

## 6. Conclusions

In recent years, the intensification of standard chemotherapy, more precise risk classification, improvements in supportive care, and the use of minimal residual disease to monitor response to therapy contributed to the improvement of the curability of AML patients in low-risk groups. Despite this, the curability of patients with high-risk AML in the pediatric population is still low. Many children become refractory or relapse even after successful therapy. A major challenge is the lack of specific antigens on the surfaces of tumor cells. Modern medicine has a wide range of treatment protocols, but only extending them with new therapeutic targets can provide a chance to improve the cure rate in high-risk groups. The increasing number of emerging clinical trials offers hope for new therapeutic solutions in the near future and improvement of the cure rate in the pediatric AML population.

## Figures and Tables

**Table 1:** Characteristics and prognosis in pediatric AML.

Molecular Alteration	Frequency in Pediatric AML	Prognosis	References
t(8;21)(q22;q22) *RUNX1::RUNX1T1*	10–12%	Very good 5-year OS 80–90%	[13,14]
inv(16)(p13.1q22) or t(16;16)(p13.1;q22); *CBFB::MYH11*	10%	Very good 5-year OS 80–90%	[13,14]
t(15;17)(q24.1;q21.2) *PML::RARA*	5–10% 2% in infants	Very good due to advanced therapy 5-year OS 95% 5-year EFS 90%	[15]
*NPM1* gene mutations	10% (50–60% in adult AML)	Very good 5-year OS 85% Better prognosis without *FLT3*/ITD	[16,17]
*CEBPA* gene double mutations	5.6% Approximately 70% of *CEBPA*_mt_ cases	Very good 5-year OS 80%	[18,19]
*CEBPA* gene single mutations	2.4%	Poor 5-year OS 25%	[18,19]
t(16;21)(q24;q22) *RUNX1::CBFA2T3*	0.2%	Good/intermediate 4-year EFS 77%	[20,21]
Trisomy 21	15%	Good Higher sensitivity to chemotherapeutic agents	[22,23]
*FLT3*/ITD mutation	Frequency increases with age 1.5% in infants 7% in children aged 1–5 17% in adolescents and young adults	Poor 5-year OS 30–40% for patients with high allelic ratios	[24,25]
11q23 (*KMT2A*) rearrangements	20% Most frequently in infants	Varies depending on *KMT2A* fusion partner	[12,26,27,28,29,30]
t(9;11)(p22;q23) *KMT2A::AF9(MLLT3)*	6–9%	Intermediate	A/M
t(11;19)(q23;p13.1) *KMT2A::ELL*	1–2%	Intermediate	A/M
t(11;19)(q23;p13.3) *KMT2A::ENL(MLLT1)*	1–2%	Intermediate	A/M
t(10;11)(p12;q23) or ins(10;11)(p12;q23q13) *KMT2A::AF10(MLLT10)*	2–3%	Poor	A/M
t(6;11)(q27;q23) *KMT2A::AF6(MLLT4)*	1–2%	Poor	A/M
t(5;11)(q35;p15) *NUP98::NSD1*	3–4% Strong association with *FLT3*-ITD	Poor 4-year EFS below 10%	[31,32,33]
t(11;12)(p15;p13) *NUP98::KMD5A*	1–2%	Poor 5-year OS 33%	[34,35,36]
t(7;12)(q36;p13) *MNX1::ETV6*	Below 30% Only in infants (<2 y.)	Poor 3-year EFS below 24%	[24,37]
Monosomy 7	4%	Poor 10-year OS 32% 10-year EFS 29%	[13]
Monosomy 5/del(5q)	1.2%	Poor 5-yer OS 27% 5-year EFS 23%	[38]
t(6;9)(p22;q34) *DEK::NUP214*	1.2–4%	Poor 5–10 year OS 30–40% 10-year EFS 30%	[13,38]
inv(16)(p13.3;q24.3) *CBFA2T3::GLIS2*	2%	Poor 5-year EFS 25–30%	[39]
inv(3)(q21q26.2) or t(3;3)(q21;q26.2) *EVI1* (former *MECOM*)	1–2%	Poor Long-term OS < 10%	[11,40,41]
t(16;21)(p11;q22) *FUS::ERG*	0.4%	Poor 4-year EFS 7%	[20,21]
t(9;22)(q34;q11) *BCR::ABL1*	1% in adults	Uncertain significance Mainly depends on coexisting aberrations	[42,43]
t(10;11)(p12;q14) *PICALM::MLLT10*	<1%	Uncertain significance	[21,44]
t(8;16)(p11;p13) *MYST3::CREBBP*	10%	Spontaneous remission has been observed	[24,45,46]
*KIT* gene mutations	5% overall 25% in CBF-AML population	Uncertain significance May negatively impact response to therapy	[47,48]
*FLT3*/TKD mutations	7% inv(16) AML	Uncertain significance	[49]
t(1;22)(p13;q13) *RBM15::MKL1*	0.3%	Uncertain significance 5-year EFS 54.5%, 5-year OS 58.2%	[35,50,51]
t(3;5)(q25;q35 *NPM1::MLF1*	<0.5%	Uncertain significance	[42,52,53]
Trisomy 8	3%	Uncertain/discussed significance 5-year EFS 25%	[13,54,55]
Hyperdiploid karyotype	11%	Uncertain significance	[56,57]
Complex karyotype	8–15%	Uncertain/discussed significance	[13,14]

Abbreviations: AML: acute myeloid leukemia; CBF: core binding factor; EFS: event free survival; ITD: internal tandem duplication; OS: overall survival; TKD: tyrosine kinase domain.

**Table 2:** Differences in adult and pediatric AML genetics.

Molecular Alteration	Pediatric AML			Adult AML		
	Frequency	Prognosis	References	Frequency	Prognosis	References
t(8;21)(q22;q22) *RUNX1::RUNX1T1*	10–12%	Very good	[13,14]	3.5%	Good	[11]
inv(16)(p13.1q22) or t(16;16)(p13.1;q22) *CBFB::MYH11*	10%	Very good	[13,14]	3%	Good	[11]
t(15;17)(q24.1;q21.2) *PML::RARA*	5–10% 2% in infants	Very good due to advanced therapy	[15]	6%	Good	[11]
*NPM1* gene mutations	10%	Very good Better prognosis without *FLT3*/ITD	[16,17]	50–60%	Very good	[58]
*CEBPA* gene double mutations	5.6%	Very good	[18,19]	5%	Very good	[59,60]
*CEBPA* gene single mutations	2.4%	Poor	[18,19]	2.3%	Poor	[59,60]
*FLT3*/ITD mutation	Frequency increases with age 1.5–17%	Poor	[24,25]	20–35%	Good/intermediate	[25,61]
11q23 (*KMT2A*) rearrangements	20%	Mostly intermediate and poor	[12,26,27,28,29,30]	15–20%	Mostly intermediate and poor	[62]
t(6;9)(p22;q34) *DEK::NUP214*	1.2–4%	Poor	[13,38]	0.5%	Poor	[11]
t(9;22)(q34;q11) *BCR::ABL1*	The vast majority of adult cases	Uncertain	[42,43]	1%	Uncertain	[42,43]
t(8;16)(p11;p13) *MYST3::CREBBP*	10%	Uncertain	[24,45,46]	0.2–0.4%	Uncertain	[63]
*KIT* gene mutations	5%	Uncertain	[47,48]	12.8–46.1% in CBF leukemia	Uncertain	[64]
*FLT3*/TKD mutations	7% in inv(16) AML	Uncertain	[49]	28% in inv(16) AML	Uncertain	[49]
Trisomy 8	11%	Uncertain	[13,54,55]	5%	Uncertain	[11]
Complex karyotype	8–15%	Uncertain	[13,14]	14%	Uncertain	[11]
t(11;12)(p15;p13) *NUP98::KMD5A*	1–2%	Poor	[34,35,36]	Lesions typical of pediatric AML		
inv(16)(p13.3;q24.3) *CBFA2T3::GLIS2*	2%	Poor	[39]			
t(7;12)(q36;p13) *MNX1::ETV6*	Below 30%	Poor	[24,37]			
inv(3)(q21q26.2) or t(3;3)(q21;q26.2) *EVI1* (former *MECOM*)	1–2%	Poor	[11,40,41]			
t(1;22)(p13;q13) *RBM15::MKL1*	0.3%	Uncertain	[35,50,51]			
t(3;5)(q25;q35 *NPM1::MLF1*	<0.5%	Uncertain	[42,52,53]			

Abbreviations: AML: acute myeloid leukemia; CBF: core binding factor; EFS: event free survival; ITD: internal tandem duplication; TKD: tyrosine kinase domain.

## Data Availability

No new data were created or analyzed in this study. Data sharing is not applicable to this article.
